# Behaviour change practices in exercise referral schemes: developing realist programme theory of implementation

**DOI:** 10.1186/s12913-021-06349-9

**Published:** 2021-04-13

**Authors:** John Downey, Katie Shearn, Nicola Brown, Ross Wadey, Jeff Breckon

**Affiliations:** 1 School of Sport, Health, and Wellbeing, Plymouth Marjon University, Plymouth, PL6 8BH UK; 2 Centre for Health and Social care Research, Sheffield Hallam University, Sheffield, S10 2BP UK; 3 Faculty of Sport, Applied Health and Performance Science, St Mary’s University, Twickenham, TW1 4SX UK; 4 Faculty of Health and Wellbeing (HWB) Academy of Sport and Physical Activity, Sheffield Hallam University, Sheffield, S10 2BP UK

**Keywords:** Implementation, Primary care, Mechanisms of action, Knowledge translation, Physical activity, Behaviour change

## Abstract

**Background:**

Exercise Referral Schemes have been delivered worldwide in developed countries to augment physical activity levels in sedentary patients with a range of health issues, despite their utility being questioned. Understanding the implementation mechanisms of behaviour change practices is important to avoid inappropriate decommissioning and support future service planning. The aim of this study was to develop initial theories to understand what influences the behaviour change practices of Exercise Referral practitioners within the United Kingdom.

**Methods:**

An eight-month focused ethnography was undertaken, to carry out the first phase of a realist evaluation, which included participant observation, interviews, document analysis, and reflexive journaling. A comprehensive implementation framework (Consolidated Framework for Implementation Research) was adopted providing an extensive menu of determinants. Mechanisms were categorised based on the Theoretical Domains Framework (within the Capability, Opportunity, Motivation, Behaviour model) providing an explanatory tool linking the levels of the framework.

**Results:**

Three programme theories are proposed. Firstly, motivation and capability are influenced when behaviour change oriented planning and training are in place. Secondly, motivation is influenced if leadership is supportive of behaviour change practice. Lastly, integration between health professionals and practitioners will influence motivation and capability. The conditions necessary to influence motivation and capability include a person-centred climate, cognizant practitioners, and established communities of practice.

**Conclusions:**

The findings are the first to articulate the necessary elements for the implementation of behaviour change practices in Exercise Referral services. These results outline emerging theories about the conditions, resources, and explanations of behaviour change implementation that can inform service development.

**Supplementary Information:**

The online version contains supplementary material available at 10.1186/s12913-021-06349-9.

## Background

Physical activity (PA) participation has an inverse relationship with all-cause mortality and is therapeutic for a range of diseases [1]. Exercise Referral Schemes (ERSs) are a primary care service used extensively in the USA, Canada, across Europe, New Zealand, and Australia [2]. Schemes typically involve inactive patients, who present with an array of chronic diseases, who are referred by a health professional to an exercise specialist for a time limited PA programme [3].

ERSs evolved organically and their utilisation across Europe expanded rapidly. The accelerated scale up, within the United Kingdom (UK), means practice is typically atheoretical, diverse, and lacks common operating systems [4]. Reviews of ERSs conclude that the service offers negligible benefit compared to brief PA advice. Additionally, it is noted that evidence is constrained by the heterogeneity of programme provisions and poor evaluative practices [5, 6].

Swedish ERSs have achieved superior outcomes in comparison to the UK [2, 7]. Of interest in Swedish literature is the explicit planning for behaviour change practices. In Sweden it is recommended that schemes examine the implementation context to support the motivation and capability of practitioners to use behaviour change strategies [8]. In the UK, it is also recommended that ERSs adopt behaviour change techniques, theory led practice, and person centred care to create an empowering and motivating environment [9]. The UK recommendations, however, provide no guidance on how to support the implementation of practice, despite calls to utilise determinant frameworks to build configurations of how context influences knowledge translation [10]. Subsequently recent ERS research has illustrated inadequate behaviour change implementation whereas the factors which influence practice are underexplored [11].

The reliance on systematic reviews fails to explain how services achieve their outcomes and many behaviour change services may be mislabelled as ineffective when the delivery of the interventions may be inadequate not the programme design [12]. Furthermore, more broadly, there is a lack of understanding of how behaviour change practices are utilised in primary care and the conditions that support implementation. If ERSs can be appraised in a valid manner research is needed to understand how context impacts evidence implementation and the processes that influence practitioner behaviour [13], Therefore, the aim of this research was to build initial ideas about how behaviour change practices are implemented in ERSs in real time naturalistic settings.

## Methods

### Approach

Realist evaluation (RE) was adopted as it moves beyond the description of an intervention to explain what works, for whom, in what context and why [14]. Realism suggests that hidden mechanisms, which operate through collective human decision making influenced by social conditions, have with the potential to cause outcomes that are observable [15]. Individual experience, however, is constructed and although accounts can be more or less true they are always fallible [16]. Interpretations will be an example of something, and the use of theoretical and conceptual frameworks is encouraged to redescribe data using established constructs. Realist evaluators attempt to explain patterns of human behaviour, in this case the presence/absence of behaviour change practices by practitioners (outcomes), by building and testing theories of how resources impact reasoning (mechanisms) and what under what conditions (context). The primary phase of a RE is the generation of programme theories, which are the envisaged causal workings of a programme. Focused ethnography privileges shared experiences by those within subcultures and typically focuses on understanding a cultural perspective on a specific problem [17]. Focused ethnography was therefore used, within a realist evaluation, to obtain immersion in the local context and co-create programme theory with actors about how behaviour change practices are implemented in ERSs.

### Researcher characteristics

The lead author is a male lecturer with 5 years of applied work across health promotion. The lead author has a background in nutrition and preventive cardiology which is more aligned to positivism, however, this work is part of a PhD and extensive mentoring and training have been undertaken within realist evaluation and ethnography. There were presuppositions about behaviour change practice, primary care culture, and staff competencies that drew the lead author to this topic. In practice these were acknowledged and challenged through informal interviews and reflexive journaling.

### Study setting

The focused ethnography took place over eight months within an ERS which had three different programmes. The ERS was in a borough of London in England that covered areas of high and low socioeconomic status. The service included a central administrative hub that organised the referrals from health professionals. The service was a 12-week gym programme with one free session a week and subsidised entry for other sessions. The structure involved an initial assessment at baseline and end point follow up.

The first author undertook all the data collection including fieldwork (245 h), working at one site weekly and visiting the other schemes bi-monthly, interviews, observations, and data analysis. KS also provided a secondary analysis of the realist statements during the analysis. Fieldwork involved working at the leisure centre or gym space and interviews were undertaken in the personal offices of staff.

One of the programmes was based at the university and two participants knew the lead author due to their previous studies. All of the other participants did not know the research team and only knew of the broad research goals, what was required from them, and the rationale for their involvement.

### Sampling

The service was purposefully sampled as purposive work (conversations, attending ERS training, past experiences, literature review) envisaged that the service format, staffing structure and diversity of schemes would be examples of mechanisms in action. Documents from national guidance, quality frameworks, ERS toolkits, and local programme specifications were purposefully sampled based on their relevance and ability to explain implementation (*n* = 13) (see Table 1). All frontline practitioners (*n* = 8) were formally interviewed during the last month of fieldwork. The formal interviews occurred once and were in addition to spontaneous and purposeful informal interviews and participant observation to discuss, and experience, emerging elements of theory throughout the study period.

**Table 1:** Overview of the documents included in the document analysis

Documents	Author
Exercise referral systems: a national quality assurance framework	Department of Health (2001)
Professional and operational standards for exercise referral	Joint Consultation Forum (2011)
A toolkit for the design implementation & evaluation of exercise referral schemes: guidance for exercise professionals	British Heart Foundation Centre for Physical Activity and Health (2010)
Physical activity: exercise referral schemes	National Institute for Health and Care Excellence (2014)
Behaviour change general approaches	National Institute for Health and Care Excellence (2007)
A toolkit for the design, implementation & evaluation of exercise referral schemes	British Heart Foundation Centre for Physical Activity and Health (2010)
Exercise for life physical activity in health and disease	Royal College of Physicians (2012)
Exercise referral exercise instructor qualification training manual	Training provider 1 (2015)
Service specification lifestyle prevention service	Council 1 (2015)
Service specification exercise referral	Council 1 (2015)
Sporting future a new strategy for an active nation	Sport England (2016)
Global action plan on physical activity 2018–2030	World Health organisation (2018)
Blueprint for an active Britain	Ukactive (2014)

### Ethics

Ethical approval was granted by the University ethics panel. All data collection was overt and verbal consent was obtained to observe participants whilst written consent was gathered for the formal interviews. All data was managed in line with legislation and in a confidential manner.

### Data collection methods

Document analysis, participant observation, reflexive journaling, and interviewing were adopted during the focused ethnography. The analysis of the documents, and early journaling and memos, provided the candidate theories that were then refined through further fieldwork and the formal interviews.

The document analysis was used to glean behaviour change definitions, envisaged resources to support implementation, and potential explanations of implementation. Specific websites were searched using the phrases ‘exercise referral’, ‘physical activity prescription’, ‘exercise on referral’, ‘physical activity promotion’ (Google, Google scholar, Register for Exercise Professionals, Sport England, local authority databases). Local documentation, including the programme tender specification and programme delivery protocols, were also analysed to identify local interpretations of national recommendations.

The observation approach undertaken included complete participant, participant as an observer, observer as a participant [18] due to the nature of the service which was isolated and dynamic. Service users were present in the culture, however, the focus on implementation explored the perceptions of staff and how contextual factors influences implementation so patients were not involved in data collection. Reflexive journaling retrospectively thought about experiences, considered thoughts and feelings about the experiences, and proposed emerging ideas about implementation. Memos were used as an analytic platform to theorise the potential links between concepts, describe data through a theoretical lens, and propose the essential conditions needed for implementation to exist. The memos were undertaken with the core questions ‘what makes this possible?’ and ‘what must reality be like for this to be the case’.

Semi-structured realist interviews (68.5 ± 12 mins) were undertaken with all frontline practitioners. All practitioners were invited to participate in the interviews either in person or by email and all agreed to take part. All interviews were carried out in their place of work at a time convenient to them and were audio recorded and transcribed verbatim. The interview adopted a teacher-learner approach whereby the researcher teaches the interviewee about the proposed causal theory and then the interviewee, from an informed position, can teach the researcher about their experience of the theory in action [19]. A topic guide (see Supplementary file 1) was produced based on the document analysis and fieldwork to propose and refine initial candidate theories with the participants. The topic guide had minor amendments after the first two interviews to support conversational theorising.

### Data analysis

The context, mechanism, outcome (CMO) heuristic was used as a thematic analysis tool to build realist programme theory. The CMO heuristic was used to link where implementation outcomes were discussed using casual language and the contextual factors associated with this. The Consolidated Framework for Implementation Research (CFIR) then provided a coding manual to abstract data to established constructs across the levels of the implementation system [20]. The Capability, Opportunity, Motivation Behaviour model (COM-B), and associated Theoretical Domains Framework, were combined with the CFIR providing expanded constructs related to individual behaviour and an explanatory apparatus to theorise casual links across the levels of the system [21].

#### Phase 1

Initially all the documents (Table 1) were uploaded to NVivo 10. Documents were read and re-read to become familiar with the content before a specific search for aspects that discussed behaviour change and implementation were sought. During the document analysis, initial journal entries and memos were also uploaded to NVivo 10 providing linked aspects of context to the emerging programme theories. Inductive codes were generated for large passages of text where aspects of implementation were discussed. Memos from reading the documents, and relevant fieldwork, were linked to the inductive codes generating broad theory areas. The CMO heuristic was then used, within these theory areas, to build configurations of how resources were envisaged to impact practitioner implementation and the potential conditions needed for this to occur. Once the theory areas were arranged in CMOs, deductive analysis was undertaken using the a priori implementation frameworks to re-describe data using theoretical labels.

#### Phase 2

Subsequent journal entries, memos, and interviews were then uploaded to NVivo 10 to refine the candidate theories. The theory areas, developed from phase 1, provided categorising codes for large passages of text to be organised. Once all the additional data was separated into a broad theory area the CMO heuristic was again used to build programme theory. The coding frameworks were then applied in a deductive manner. Lastly, each CMO statement within the data was checked to see if it corroborated, refuted, or refined previous programme theory. If novel insight was drawn, new emerging theories were formed and if there was crossover between other statements the theory was augmented to encompass the core characteristics of each CMO.

### Rigour

Research quality was conceptualised from a realist viewpoint [22]. The use of multiple data strands, co-created theory building, and extensive journaling was adopted to enhance the descriptive validity and transparency of the work. The adoption of the CMO heuristic, fieldwork, and use of established theoretical frameworks attempted to enhance the plausibility of causal ideas. Lastly the use of focused ethnography and ongoing theory adjudication, from the onset, makes the work more acceptable and useful for practical recommendations. Datasets were coded by the lead author and circulated to the research team. The lead author also presented his findings to the rest of the team to act as critical friends and discuss potential alternative interpretations of the CMOs.

## Results

**Fig. 1:**
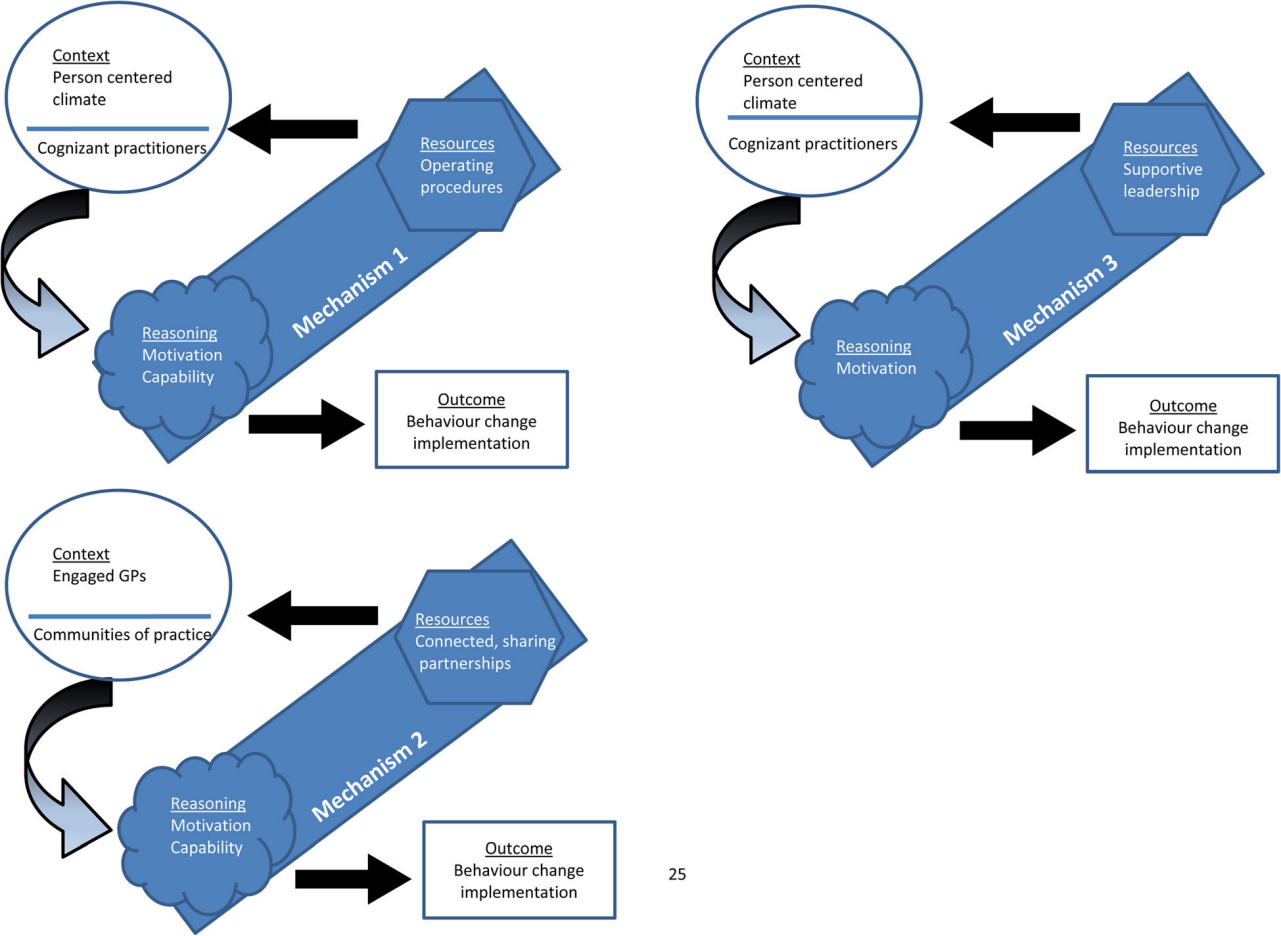
Visual representation of the programme theories using Dalkin and colleagues CMO templates [23]

The formal interviews are the only data presented below as they verified and elaborated the theories from other data sources including the documents and journal. ‘Behaviour change consistent operating procedures’, ‘integration with medical professionals’ and ‘supportive leadership’ are proposed to modify practitioner capability and motivation to implement behaviour change practices (Fig. 1). Motivation and capability are broad mechanisms which encompass 12 domains of theoretical constructs (see [24]. The mechanisms are represented using motivation and capability labels to enhance the transferability of the findings to other implementation scenarios and encompass multiple interpretations from practitioners (Fig. 1). These mechanism labels are broken down across the 12 domains in the results presented below under the programme theory sub-headings.

The document analysis and initial reflections produced over 70 potential activities related to implementation across four theory areas and 30 potential mechanisms of action. Iterative theory building through field notes, observations, and interviewing led to the consolidation of two theory areas, removal of one, and inclusion of another. What is presented below is the cumulation of enduring facets that emerged across all data collection procedures.

### Programme theory 1: behaviour change consistent operating procedures

Operating procedures was a broad term used by the authors to package provisions which demonstrated organisational planning to support behaviour change practice. Training, mentoring, appraisals, feedback loops, and ongoing CPD were consistently referenced to by practitioners as essential operating procedures for implementation. The provisions were postulated to impact skills, knowledge, and the memory capabilities of practitioners. Alongside changes to capability, training was suggested by practitioners to influence motivation via identity and automatic motivation processes.

Additionally, practitioners highlighted that behaviour change protocols including practice manuals, service plans, and resources for behaviour change interactions were important for implementation. Practitioners highlighted that the lack of behaviour change protocols impacted upon their motivation to use behaviour change practices due to a diminished role clarity. In addition, the lack of behaviour change focused procedures altered their intentions to use behaviour change practices. The following quote highlights that despite a desire to practice behaviour change a lack of organisational planning created difficulties when trying to implement them.

*Em, yeah maybe like what is normal for us to do in practice, what is talked about because as I said before it is only really my norm to think about these things because we learned it at uni … .. There are a lack of strategies to help tackle the situation where behaviour change is not working as well, there wasn’t any strategy in place to say ‘ok well we need to actually focus on this for every person and if this happens what is the way around it’. That would empower me to be there at my job and focus on behaviour change (Interviewee 1).*

The absence of generic organisational planning and practice expectations had consequences which did allow those who were cognizant and driven to support behaviour change to act on their own motives. The quote below describes how the lack of organisational pressure and freedom allowed some practitioners to practice as they wished along with the implications of attending training.

I*f you’re looking at materials and that kind of thing and behaviour change, they don’t really take that into account, they’re just looking at targets. I did an introduction to counselling skills course as well and mental health first aid, as, I thought those would be useful skills. It wasn’t anything to do with here it was just personal. I think just generally it improves your communication and listening skills … so, it improves knowledge and then you can better understand what stages people are at and what you can do to help them get on to the next stage. (Interviewee 8).*

The hypothesised motivation and capability mechanisms are therefore contingent on contextual factors. Practitioners suggested that motivation and capability would become more widespread in response to behaviour change planning and training if: i) a person-centred climate existed, ii) practitioners were cognizant of behaviour change practices and iii) practitioners had a desire to support individual independence. Many practitioners are personal trainers, yet, ERSs were seen as incongruent with personal training which is highlighted in the following quote.

*I think the way PTs (personal trainers) operate doesn't really facilitate for behaviour change to be used; they don't care, they don't want to listen to their client, like they are there to train them. I think it's very different, PTs I think tend to want the client to rely fully on them for their changing behaviour (Interviewee 2).*

In brief, if there is a person-centred climate and cognizant practitioners (C) and the service provides behaviour change orientated planning and training, then practitioner’s motivation and capability (M) will increase leading to the implementation of behaviour change practices (O).

### Programme theory 2: integration with medical professionals

Practitioners explained that integration with General Practitioners (GPs)/ referrers through phone calls, email, documentation, meetings, role modelling, and vicarious learning influences their motivation and capability. Integration was thought to influence motivation through changes in emotions, group identity, and intentions. Integration was also suggested to increase the ability to self- regulate behaviour change practices in which conscious efforts were made to build/break habits. One practitioner described their contrasting roles and how integration impacted their motivation to implement behaviour change practices.

*But it was working remotely so again it was like a very independent role, I was on my own in the leisure centres delivering, whereas in the NHS you had a set structure and you had people to consult with like to bounce ideas off it was so much more motivating like when you were in a team to like enhance your practice (Interviewee 2).*

It is suggested that integration would influence the motivation of practitioners when GPs understand, value, and are interested in ERSs and behaviour change, and when there is a perceived shared effort across professions. The below quote illustrates what conditions are needed if integration is to work.

*If the GPs in the area knew what the skills of the staff here had, that could make quite a big difference … I think, erm, yeah you get some inappropriate referrals and you kind of think if I just spoke to the GP I could find out what the correct thing is for the patient really … help being validated as a profession and then on the upside if I feel the GPs are buying into it, I feel a little bit more like ‘ah, this is what we’re supposed to be doing’ (Interviewee 8).*

The realist theory is as follows, if there is GP enthusiasm for ERS, and a perceived connected team effort for behaviour change (C), and there are established integrative communication channels, then practitioner motivation and capability will increase (M) leading to the implementation of behaviour change practices (O).

### Programme theory 3: supportive leadership

The analysis highlighted that supportive leadership through role modelling, clear behaviour change expectations, and the allocation of resources to improve the practice of behaviour change was important. The provision of supportive leadership activities is suggested to influence motivation through identity, intentions, and the emotions of practitioners, illustrated in the following quote.

*Well, with here there was buy in throughout the chain whereas in other places there’s not, not even from the upper management in other places. Ms S is great for this because she was really, she had the same motivation as me, but she had quite an in-depth knowledge on it (behaviour change) and she mentioned she was getting trained in behaviour change so, a mentor would be great for people with no experience … . to weirdly enough instil a behaviour change in you a self-fulfilling prophecy, you know (Interviewee 5*).

Nonetheless, the impact of leadership activities on motivation were often contingent on a person-centred climate and behaviour change cognizant practitioners. Where the service focuses on throughput as a measure of success, and practitioners were unaware of their role within behaviour change, practitioners outlined that self-preservation would impinge their ability to implement what a leader may be trying to mobilise throughout a service. In summary, if there is a person-centred climate and cognizant practitioners (C) and supportive leadership is present, then the motivation of practitioners will improve (M), leading to the implementation of behaviour change practices (O).

## Discussion

Behaviour change orientated operating procedures, supportive leadership and integration between ERSs and medical professionals are broad themes which are suggested to influence practitioner motivation and capability. Changes to the motivation and capability of practitioners is contingent on several conditions. There must be an organisational person-centred climate, practitioners must be cognizant of behaviour change, and/or there must be a community of practice including medical professionals.

ERS literature on implementation is sparse, however, lessons from other behaviour change services can be drawn upon. Moreover, by abstracting data to established aspects of grand theory it allows the programme theories to be portable to other primary care services [25]. The current theories provide a framework and accumulation of understanding on how behaviour change science may be implemented into healthcare and thus direction for testing in other settings. What is evident across other primary care services is that similar contextual factors impinge the translation of policy to practice [26].

In this study, operating procedures that prioritise behaviour change practice, through planning and training, are suggested to influence the motivation and capability of practitioners. Operating procedures are only thought to positively impact implementation if there is a person-centred climate or if there is freedom for motivated staff to act on their own values. The need to have explicit planning and procedures for the service aim, and provide ongoing training, as outlined in this study, is supported by other research examining the implementation of behavioural interventions [27]. Additionally, when the climate adopts a focus on throughput alone, practitioner implementation of person led care is known to be impinged, confirming the data from this research [28]. The current study advances the literature by proposing tentative causal explanations of how the organisational climate and operating procedures influence practitioner behaviour.

Leadership which engages with behaviour change practice was found to impact practitioner motivation in this research. Specifically, the presence of a leader who is committed, knowledgeable, and expects behaviour change is proposed to positively impact motivation. The role of supportive leaders in evidence implementation is well established [29], however, the determinants and moderators of how leadership influences implementation is underdeveloped [30]. The current study adds to the literature on how leadership activities influence implementation by explaining how motivation is augmented through supportive leadership.

Regardless of the operating procedures and leadership provisions, or the wider person-centred climate, it was found in this study that practitioner characteristics impact behaviour change implementation. It was noted that many practitioners are personal trainers and may possess a lack of self-awareness, incongruent motives, and low self-compassion which may impact their practice of behaviour change, but also their response to service planning, training, and supportive leadership [31]. Alternatively, it was shown in this study that some practitioners were able to overcome the lack of behaviour change focus and engage in self-development for behaviour change when they were cognizant, driven, and had the freedom to act on their motives.

Lastly, integration with established communication channels between medical professionals and practitioners was found to influence the motivation and capability of practitioners to implement behaviour change practices in this research. Integration augments motivation when there is a community of practice for behaviour change and practitioners perceive that medical professionals’ value ERSs according to the findings from this research. The need for team networks and integration is known to augment motivation in practitioners and support evidence based practice [32]. Communities of practice refer to a group of people who all have an authentic interest in a similar problem and interact to learn from each other [33]. Alternatively a lack of communication with GPs, GP scepticism and lack of interest in a programme, and limited shared ownership, decreases implementation [34].

## Conclusion

This study has generated initial explanatory theories of how behaviour change practices are implemented in ERSs based on lived experience. Three priority theory areas were developed and have been substantiated by contemporary implementation literature. This work advances the field by linking the conditions and resources to theoretical explanations of implementation behaviour. These programme theories were redescribed using an established implementation conceptual framework and behavioural theory and as such are portable to other areas of healthcare. The initial theories are tentative and require empirical testing in future work but provide direction for sampling which is needed to refute, augment, and validate these findings.

## Supplementary Information

**Additional file 1.** Interview topic guide.

## Data Availability

The datasets used and/or analysed during the current study are available from the corresponding author on reasonable request.

## Abbreviations

PA: Physical activity
ERS: Exercise referral scheme
UK: United Kingdom
RE: Realist evaluation
CMO: Context mechanism outcome
COM-B: Capability opportunity motivation- behaviour
CFIR: Consolidated framework for implementation research
GP: General practitioner
