# Supplementary material for: Behaviour change practices in exercise referral schemes: developing realist programme theory of implementation
Source: BMC Health Serv Res. 2021 Apr 13;21:335. doi: 10.1186/s12913-021-06349-9 (PMC8045303; doi:10.1186/s12913-021-06349-9)
Supplement: Supplementary file 1 — Additional file 1. Interview topic guide. [file 12913_2021_6349_MOESM1_ESM.docx]

Interview topic guide

*Introduction-* The approach I am taking works by both of us actively discussing the service in relation to how it impacts practitioners using behaviour change practices. I am looking for your experience within the scheme to identify what may impact the implementation of behaviour change science and the circumstances that allow this to happen. You have the local expertise that I would like to draw out in relation to how the service works, and when it works/does not work. I will pose ideas that I would like you to comment on and go beyond your immediate experience to think about how it would work for others in the scheme.

| 1. Can you tell me about your background and involvement in exercise referral?  -What is your role now and what does it involve?  2. How do you think behaviour change fits in to your role/remit?  -What are some of the difficulties that can alter behaviour change use?  -How does it sit within your workload?  3. What, in your view, does behaviour change practice for staff involve?  -What does successful behaviour change practice look like to you?  -What are the expected benefits of using behaviour change practices? |
| --- |
| 4. I am curious about how things cause behaviour change (lack of) implementation. Thinking about your practice what do you think impacts your use of behaviour change?  -What did (element named) provide that can impact practice? Has the (element named) changed the way you feel or think? Would this be the same for all staff? If not why not?  -Is there other circumstances where this would not be the case? |
| 5. There are lots of ideas about how behaviour change implementation works and it is likely that it works differently, in different places, and for different people. One of the ideas is (mechanism/theory area posed to individuals who has potential to provide insight). Has it worked like that for you?  -Can you give an example? Why and how does it work like this?  -Can you think of other circumstances where this may play out? How would this impact on staff’s responses?  6. When (theory area) has been experienced in your practice, what has allowed this to happen and what affect did it have on staff? Why was this the case? Where their some staff who responded differently? Why do you think this was the case?  7. Thinking about some of the ideas we discussed, is there one area that you see as crucial without it nothing else would work for implementation of behaviour change?  8. Are there any other reasons why you think staff use behaviour change techniques that we haven't covered today?  9. If you could change something about the service/people/networks to make it work more effectively what would it be and why? |
